# Exploring physicians, nurses and ward-based pharmacists working relationships in a Swedish inpatient setting: a mixed methods study

**DOI:** 10.1007/s11096-019-00812-8

**Published:** 2019-04-01

**Authors:** Marcia Håkansson Lindqvist, Maria Gustafsson, Gisselle Gallego

**Affiliations:** 1 Department of Education, Mid Sweden University, 851 70 Sundsvall, Sweden; 2 Department of Pharmacology and Clinical Neuroscience, Umeå University, 90187 Umeå, Sweden; 3 Present Address: School of Medicine, The University of Notre Dame Australia, Sydney, NSW Australia

**Keywords:** Clinical pharmacy, Collaboration, Nurses, Physicians, Sweden

## Abstract

*Background* In Sweden there has been limited work investigating the integration and nature of collaborative relationships between pharmacists and other healthcare practitioners. *Objective* To explore the working relationships of physicians, nurses and ward-based pharmacists in a rural hospital after the introduction of a clinical pharmacy service. *Setting* General medical ward in a rural hospital in northern Sweden. *Method* Mixed methods involving face-to-face semi-structured interviews with nurses, physicians and pharmacists, and a physician survey using the Physician-Pharmacist Collaboration Index to measure the extent of physician-reported collaborative working relationships. *Main outcome measure* Perceptions about collaborative working relationships between physician, nurses and pharmacists. *Results* All physicians (n = 9) who interacted with the clinical pharmacists completed the survey. The mean total score was 78.6 ± 4.7, total 92 (higher scores represent a more advanced relationship). Mean domain scores were highest for relationship initiation (13.0 ± 1.3, total 15), and trustworthiness (38.9 ± 3.4, total 42), followed by role specification (26.3 ± 2.6, total 30). The interviews (with nurses and physicians), showed how communication, collaboration and joint knowledge-exchange in the intervention changed and developed over time. *Conclusion* This study provides new insights into collaborative working relationships from the perspectives of physicians and nurses. The Physician-Pharmacist Collaboration Index scores suggest that physicians felt that clinical pharmacists were active in providing patient care; could be trusted to follow up on recommendations; and were credible. The interviews suggest that the team-based intervention provided good conditions for creating new ways to work to achieve commitment to professional working relationships.

## Impact on practice

- For pharmacists, training with other professions such as physicians and nurses may provide possibilities to improve interprofessional collaborative care.
- For physicians, nurses and pharmacists improved interprofessional collaborative care may lead to job satisfaction as well as an understanding of the responsibilities of other professionals in their professional roles.
- Improved interprofessional collaborative care may also lead to improved patient safety.

## Introduction

Today, many pharmacists are involved in clinical pharmacy services in hospital wards, and in many places, pharmacists have become integral members of the ward teams [1, 2]. The European Statements of Hospital Pharmacy state that the overarching role of a hospital pharmacy is to optimise patient outcomes through working collaboratively within multidisciplinary teams to achieve the responsible use of medicines [3]. The importance of interdisciplinary collaboration has increased, and it has been found that collaborative care between pharmacists, physicians and nurses has the potential to improve patient outcomes as well as the process of care [4, 5]. Therefore, it is important to understand how a collaborative relationship between physicians, nurses and a ward-based pharmacist develops.

To assist researchers and practitioners interested in pharmacist collaboration, a theoretical framework for physician/pharmacist collaborative working relationships (CWR) has been proposed [6]. In this model, professional working relationships are categorized into five progressive stages from Stage 0 (professional awareness) to Stage 4 (commitment to professional working relationship). The framework also describes three factors that drive the development of the collaborative relationship: participant, context and exchange characteristics. Level of education and training experience are examples of participant characteristics, while context characteristics are related more to the environment such as organizational structure. Exchange characteristics cover the social exchanges between pharmacists and physicians [6].

Relatively little work has been done in Sweden to investigate the integration and nature of CWRs between pharmacists and other healthcare practitioners such as physicians and nurses [7, 8]. In Swedish hospitals, clinical pharmacists have not been traditionally part of the core patient care team, although the number of clinical pharmacists working in hospitals is increasing. In September 2015, a clinical pharmacy service was implemented as part of a study in a general medical ward of a hospital in northern rural Sweden. The study was conducted over a six-month period and looked at whether medication reviews performed by clinical pharmacists as part of a ward team could reduce drug-related problems (DRPs) [9]. In order to understand the expectations and perceptions of physicians and nurses, a qualitative study was conducted prior to the introduction of this service [10]. Before these interviews, clinical pharmacists had not been involved in providing patient care services at this hospital. The study found that the physicians and nurses had limited experience of working with clinical pharmacists, had limited knowledge about their clinical skills and clinical competence, and they had negative perceptions. Nurses thought that pharmacists were going to take work away, and doctors thought that their workload was going to increase. These were perceived to be barriers to a successful implementation of the service [10].

## Aim of the study

The aim of this study was to explore the working relationships of physicians, nurses and ward-based pharmacists in a rural hospital after the introduction of a clinical pharmacy service.

### Ethics Approval

The study was approved by the Regional Ethical Review Board in Umeå (Registration Number 2014/322-31Ö).

## Method

### Setting

This study was performed at a medical ward in a hospital located in northern rural Sweden. At the time of the study, the general medical ward had 18 beds. The hospital is one of three hospitals situated in Västerbotten County and is the only one located inland, providing medical services for around 40,000 people Due to its geographic location it is also the base of the ambulance helicopter [11, 12].

### The intervention

The intervention (clinical pharmacy service) was implemented in September 2015. Three clinical pharmacists provided clinical services including medication reconciliation, medication reviews and participation in ward rounds. The intervention is described in detail in Peterson et al. [9]. The service was provided three days a week for 6 months. The clinical pharmacists who provided the service had postgraduate training in clinical pharmacy and had practiced as hospital-based clinical pharmacists prior to participating in this study.

### Participant recruitment

All physicians and nurses in the ward in which the pharmacists’ intervention was implemented were invited to participate. Email invitations to participate in a face-to-face, semi-structured interview, were sent by the clinical nurse manager. Days and times were chosen to best suit the workload and staffing of the ward. All pharmacists involved in the intervention were interviewed. All interviewees were given information about the study and were informed that participation was voluntary. An interview schedule was developed with a list of topics to be discussed during the interviews, including practice environment, barriers and enablers and interactions/relationships with the pharmacists. Physicians and nurses were asked about the pharmacist’s role and contribution, and challenges of having a ward-based pharmacist.

This study employed mixed methods including individual semi-structured interviews and a physician survey. The Physician-Pharmacist Collaboration Index (PPCI) [13] was used, which is a validated instrument that measures the extent of physician-reported collaborative working relationships. Physician and nurse interviews were conducted in May 2016 at the hospital. Pharmacist interviews were conducted in January 2017 at the Department of Education at Umeå University. All interviews were conducted by author MHL, an experienced interviewer. Interviews were digitally recorded with the permission of the interviewees, transcribed and translated into English.

### Data analysis

A thematic analysis was conducted of the interview transcripts, which followed several steps [14]. Transcripts were read and coded by (MHL) and (MHL and GG) independently using both an inductive (response-based) and deductive approach (research-driven with focus on the CWR framework) [15]. These themes were then read for similarity and divergence within and across themes. Data management was supported using excel. The analysis presented in this paper focuses on collaborative working relationships between pharmacists, physicians and nurses. The PPCI scores were analysed using STATA 11.0 for Windows (StataCorp LP, College Station, TX). Descriptive statistics summarised the data.

## Results

In total, 19 physicians and nurses were invited, and 14 agreed to participate. Non-participation in the study was referred to lack of time, busy schedules and transfers to other hospitals. Nine medical physicians, five nurses and three pharmacists were interviewed. Interviews typically lasted between 20 and 40 min for the physicians and nurses, and between 45 and 60 min for the pharmacists. Five of the eight physicians were male; all nurses and pharmacists were females. Four of the physicians were consultants.

All physicians (n = 9) who interacted with the clinical pharmacists during the introduction of a clinical pharmacy service completed the Physician-Pharmacist Collaboration Index (PPCI) survey. The mean total PPCI score was 78.6 ± 4.7, total 92 (higher scores represent a more advanced relationship). Mean domain scores were highest for relationship initiation (13.0 ± 1.3, total 15), and trustworthiness (38.9 ± 3.4, total 42), followed by role specification (26.3 ± 2.6, total 30). A summary of PPCI domain and total scores is provided in Table 1.

**Table 1:** Team-based physician-rated Physician-Pharmacist Collaboration index (PPCI) domain scores (n = 9)

PPCI score (possible range)	PPCI score^a^ (mean ± SD)	Range
Total score (14–92)	78.6 ± 4.7	75–86
Domain		
Relationship initiation (3–15)	13.0 ± 1.3	11–15
Trustworthiness (6–42)^b^	38.9 ± 3.4	33–42
Role specification (5–35)	26.3 ± 2.6	22–30

^a^Higher scores represent a more advanced relationship

^b^n = 8—one physician had one missing value in the trustworthiness domain

The interviews were used to further explore the relationships between physicians, nurses and pharmacists. The CWR domains were used to initially describe the interactions. Three broad themes were identified. Within the first theme, *Initiating relationships*, two subthemes emerged: (1) *Initial expectations and apprehensions*, and (2) *Learning to collaborate*. Within the second theme, *Role specification*, three subthemes were identified: (1) *Value added*, (2) *Accessibility* and, (3) *Patient care and safety*. The third theme comprised *Barriers and enablers*. To allow the reader to judge the veracity of the interpretation, quotations have been used to illustrate the themes presented.

### Initiating relationships

In this study, the pharmacists first visited the hospital to introduce themselves and the intervention. Hence, they *initiated* the relationship with both the physicians and nurses through this presentation.

### Initial expectations and apprehensions

Pharmacists described the apprehensiveness of physicians and nurses before the intervention: “A little curiosity mixed with a little skepticism” (Pharmacist 1). It was difficult to explain their role: “We noticed that they looked confused. They wondered, what exactly we were going to do?” (Pharmacist 1). Both physicians and nurses commented on an expected focus on *drug knowledge*. Physicians had concerns about *being told what to do*. However, these concerns dissipated, being instead described as “a very humble … discussion” (Physician 3).

### Learning to collaborate

Relationships were built over time and developed through understanding the pharmacists’ contributions to the team. One physician reflected on “bringing in knowledge from different roles into the work on the rounds … so that you can have good teamwork” (Physician 7). Physicians, nurses and pharmacists built collaborative relations consistent with the CRW model; the more providers worked and communicated with each other, the more providers relied on the pharmacists’ knowledge and greater collaboration. The pharmacists reported the climate as “open-minded” (Pharmacist 2). However, relationship initiation takes time: “It takes a while before you get to know each other” (Pharmacist 1). Some physicians commented on the importance of the pharmacists’ personal traits for team collaboration as “having the right people in the position” (Physician 1).

Relationships were built over time; proximity and visibility allowed both physicians and nurses to interact and understand the pharmacists’ professional ability. Words such as *joint knowledge* and *collegiality* were used. Mutual respect developed through understanding the role and capabilities of the pharmacists as “a professional relationship in which I have great respect for their knowledge” (Physician 2).

### Role specification

#### Something to add

Most participants mentioned that pharmacists had *something to add* for physicians and nurses as well as patients. One physician noted: “You continually receive education through their comments on the rounds” (Physician 6). These issues were discussed collaboratively in the team as “a joint exchange of knowledge” (Nurse 1) in which nurses and physicians could “ask questions and discuss things” (Pharmacist 1). One physician explained: “The work with drugs is a big piece in some way, and it is missing in the teamwork” (Physician 4).

The pharmacists, nurses, and physicians exchanged information directly on the rounds “about drugs which don’t work together” (Nurse 1), which “saved us time” (Physician 4). One physician noted the pharmacists’ expertise: “We physicians have relatively little drug knowledge” (Physician 4). The professional relationships initially built during the ward rounds, continued with individual contacts with the pharmacists. These results are consistent with the relatively high score in the PCCI relationship initiation trustworthiness.

#### Accessibility

The pharmacists reported being based in the ward as positive. One pharmacist mentioned a physician who often came by to ask questions: “It takes a while before you understand what we [pharmacists] know” (Pharmacist 2). Nurses also took advantage of the pharmacists being nearby: “Nurses could come occasionally and ask something” (Pharmacist 1). One physician noted: “You could always knock on the door and ask. I have thought about this [drug]. Is this [drug] good or is there another alternative?” (Physician 4).

#### Patient care and safety

The pharmacists’ expert knowledge on side effects, speaking to patients and reviewing patients’ medication lists were seen as important. According to one physician: “They know more about drugs and we can actually help out together, this helps the patient mainly” (Physician 5). Physicians and nurses were positive about the pharmacists speaking to patients, being as “especially good if you can explain an interaction” (Physician 8). For the pharmacists, patients provided reliable information as they were “a little more open” (Pharmacist 2).

Regarding patient safety, one pharmacist reported a patient on an unsuitable drug: “With her state of illness, she really shouldn’t have been taking this” (Pharmacist 2). Another pharmacist discovered an error when a patient switched wards: “They had taken drug levels of a drug that was very neurotoxic … and the patient had already been discharged” (Pharmacist 3).

### Barriers and enablers

Overall pharmacists, physicians and nurses only identified a few barriers to the implementation of the intervention. Funding was noted: “If it is taken from the nurses’ budget, then it’s a direct no” (Nurse 5). Another barrier was time, as the rounds took longer: “Surely you can find good forms for how to do this” (Physician 6). Disagreement in decision-making was noted as a potential barrier: “In these cases I have the mandate to decide” (Physician 1). Another barrier was medication review documentation, for seeing that “the medication review has been reviewed relatively recently” (Physician 2). For the pharmacists, being based and employed at another hospital, 127 km from this rural hospital, distance was a barrier. One pharmacist reflected: “You could have been on site [at the hospital in TOWN] even more if you lived in TOWN” (Pharmacist 2).

Enablers were also reported, such as a climate at the ward which was “accepting” (Nurse 3). Another enabler was the size of the hospital: “It is smaller in [name of the place], and it is easier for you to get hold of a physician” (Pharmacist 1).

## Discussion

This study explored the working relationships of physicians, nurses and ward-based pharmacists in a rural hospital after the introduction of a clinical pharmacy service. The study found that CWR are multifaceted and involve a consideration of the professionals’ understanding of the pharmacist’s role and capabilities. As described by McDonough et al. [15], this study similarly showed the importance of understanding professional abilities. Trust is built over time, and relationships can occasionally tend to be personality-related [16]. The pharmacists involved in this study were highly-trained and experienced professionals. They described their intervention at the ward with optimistic views and reported their integration into an open, positive professional team of physicians and nurses. These conditions provided a strong basis for understanding the pharmacist’s professional abilities, for development of CWR, and supporting their work in the ward. As described by Snyder et al, individual characteristics such as educational background influence how professionals move in the collaboration progression.

Studies exploring CWR have been community-based (General Practitioners and Community pharmacists) [16, 17]. Rathbone et al.explored CWR between pharmacists and general practitioners (GPs) in Australia in the context of medication adherence [18]. The authors described that CWRs may be underpinned by trust and shared perspectives that can be constructed by their interaction during training. For a successful CWRs to occur they also needed to share similar perspectives about each other’s goals and roles. Makowsky et al. [19], explored CWR for nurses, physicians and team-based hospital pharmacists in Canada. Similarly to our results, pharmacists were highly valued, and collaborations were more successful when relationships were built on mutual trust and respect [16]. Organizational barriers (scheduling, logistics, space, employment, continuity) were also identified [19, 20].

The PPCI scores suggest that physicians felt that clinical pharmacists were active in providing patient care; could be trusted to follow up on recommendations; and were credible, but that within the domain of role specification there is room for growth of CWRs. Our PCCI scores were also similar to those found in other research studies [17, 20]. Both the PCCI scores and the qualitative data showed that in order to develop positive relationships it is important to have role clarity [17, 20]. The domain of role specification had the lowest scores, which is not surprising. As previously described, ward-based pharmacists are not commonplace in Sweden. However, this varies and in some hospitals, clinical pharmacists are well established in the ward team. Still, many physicians and nurses are unaware of the capabilities of clinical pharmacists because most pharmacists work in community pharmacies. In this role, the most common task is giving advice to the patient—not working together with other professionals.

Other factors which may affect CWR are individual, context and exchange characteristics. As noted by the participants in the study, individual characteristics do make a difference. This was mentioned by one physician with the idea of the right person in the right place for the pharmacists. From the pharmacists’ point of view this was also noted as physicians’ and nurses’ willingness to discuss, and their open-mindedness. The participants linked these conditions to patient safety and care. Communication was described as open, discussion-based and collaborative, and therefore knowledge-building, according to the participants. As previously described, the apprehension regarding knowledge experts joining the rounds was not of relevance in this study. Specifically, in this case, issues of power related to the expert role were not seen to be a problem. In this study, only female pharmacists were interviewed. A male perspective had been interesting, however, the sample reflects the gender demographics in ward-based practice in Sweden.

One of the main findings in this study related to role specification. According to this study, if pharmacists train with other professions such as physicians and nurses there may be possibilities to improve interprofessional collaborative care as well as understanding the responsibilities of other professional roles [21, 22]. In this study, this was apparent as changes in the nurses’ territorial views over time resulting from the intervention and an understanding of the pharmacists’ role.

There are several limitations that need to be considered. First, our study took place in a rural hospital where no clinical pharmacy services are provided; this provides a unique setting, where staff shortages and long distances are common. The clinical pharmacists were highly trained and experienced. Another limitation is data collection and analysis. While interviews were in Swedish, the data analysis was done in English. It is possible that certain information was lost in translation. However, most studies to date have focused on CWR between community-based pharmacists and physicians. There is less information about ward-based clinical pharmacists, physicians and nurses. To our knowledge this is the first study exploring these relationships in Sweden. The study was also longitudinal, documenting perceptions before and after implementation of the intervention, and over a long period of time (6 months). The results of the study may also be relevant in countries were ward-based pharmacists are infrequent, such as in Latin America, the Middle East, Africa and some parts of Asia and Europe.

## Conclusion

This study was able to provide new insights into CWR from the perspectives of physicians and nurses. The PPCI scores suggest that physicians felt that clinical pharmacists were active in providing patient care, could be trusted to follow up on recommendations and were credible, and that within the domain of role specification there is room for growth of collaborative relationships. The interviews with the pharmacists, physicians and nurses, in line with CWR, show how communication, collaboration and joint knowledge exchange in the team-based intervention developed over time, with a focus on patient care and safety. As the team moved from professional awareness and recognition, through exploration and trial, professional relationships expanded, providing conditions for finding and creating new ways to work to achieve commitment to professional working relationships.
